# Kruppel-like factor 4 signals through microRNA-206 to promote tumor initiation and cell survival

**DOI:** 10.1038/oncsis.2015.8

**Published:** 2015-06-08

**Authors:** C-C Lin, S B Sharma, M K Farrugia, S L McLaughlin, R J Ice, Y V Loskutov, E N Pugacheva, K M Brundage, D Chen, J M Ruppert

**Affiliations:** 1 Department of Biochemistry, West Virginia University, School of Medicine, Morgantown, WV, USA; 2 The Mary Babb Randolph Cancer Center, West Virginia University, Morgantown, WV, USA; 3 Program in Cancer Cell Biology, West Virginia University, Morgantown, WV, USA; 4 Division of Preventive Medicine, Department of Medicine, University of Alabama at Birmingham, Birmingham, AL, USA

## Abstract

Tumor cell heterogeneity poses a major hurdle in the treatment of cancer. Mammary cancer stem-like cells (MaCSCs), or tumor-initiating cells, are highly tumorigenic sub-populations that have the potential to self-renew and to differentiate. These cells are clinically important, as they display therapeutic resistance and may contribute to treatment failure and recurrence, but the signaling axes relevant to the tumorigenic phenotype are poorly defined. The zinc-finger transcription factor Kruppel-like factor 4 (KLF4) is a pluripotency mediator that is enriched in MaCSCs. KLF4 promotes RAS-extracellular signal-regulated kinase pathway activity and tumor cell survival in triple-negative breast cancer (TNBC) cells. In this study, we found that both *KLF4* and a downstream effector, microRNA-206 (miR-206), are selectively enriched in the MaCSC fractions of cultured human TNBC cell lines, as well as in the aldehyde dehydrogenase-high MaCSC sub-population of cells derived from xenografted human mammary carcinomas. The suppression of endogenous KLF4 or miR-206 activities abrogated cell survival and *in vivo* tumor initiation, despite having only subtle effects on MaCSC abundance. Using a combinatorial approach that included *in silico* as well as loss- and gain-of-function *in vitro* assays, we identified miR-206-mediated repression of the pro-apoptotic molecules programmed cell death 4 (*PDCD4*) and connexin 43 (*CX43/GJA1*). Depletion of either of these two miR-206-regulated transcripts promoted resistance to anoikis, a prominent feature of CSCs, but did not consistently alter MaCSC abundance. Consistent with increased levels of miR-206 in MaCSCs, the expression of both PDCD4 and CX43 was suppressed in these cells relative to control cells. These results identify miR-206 as an effector of KLF4-mediated prosurvival signaling in MaCSCs through repression of *PDCD4* and *CX43*. Consequently, our study suggests that a pluripotency factor exerts prosurvival signaling in MaCSCs, and that antagonism of KLF4-miR-206 signaling may selectively target the MaCSC niche in TNBC.

## Introduction

Compelling experimental evidence supports the hierarchical organization of certain human tumor types, including breast cancer.^1, 2, 3, 4, 5, 6^ These tumors comprise heterogenous mixtures of tumor cell populations that include cancer stem-like cells (CSCs), typically defined by their ability to initiate tumors in limiting dilution assays (that is, tumor-initiating cells, TICs). Furthermore, CSCs can survive and form tumorspheres in suspension culture, self-renew and differentiate.^7, 8^ These cells display resistance to chemotherapy, radiation therapy and other triggers of cell death, and are thought to contribute to cancer recurrence. Therefore, CSCs represent an important sub-population for therapeutic targeting.^3, 5^

In mammary carcinoma, functionally validated CSCs (termed MaCSCs (mammary CSCs)) have been identified by profiling the expression of cell surface markers such as PROCR (P) and ESA (E) and/or by assaying aldehyde dehydrogenase (ALDH) activity.^9, 10, 11, 12^ Despite this insight, the underlying mechanisms that mediate the MaCSC phenotype are unclear. For regulation of their abundance and/or their intrinsic properties such as resistance to cell death, several cellular signaling axes have been implicated including the WNT, NOTCH, TGFβ and SHH pathways.^13, 14, 15^

A potential mediator of the MaCSC phenotype is the pluripotency factor Kruppel-like factor 4 (KLF4). This zinc-finger transcription factor promotes the formation of induced pluripotent stem cells from adult somatic cells and can have both antitumorigenic and protumorigenic roles in a context-dependent manner.^16, 17, 18, 19, 20^ The capability of KLF4 to exert protumorigenic influences may reflect its role as a prosurvival stress response factor.^21, 22, 23, 24, 25, 26, 27, 28^ In support of a protumorigenic role, KLF4 promotes epithelial transformation *in vitro*, escape from RAS-induced senescence and skin tumor initiation in transgenic mice.^16, 29, 30^ Furthermore, loss-of-function studies reveal that KLF4 promotes cell survival following radiation-induced DNA damage, and promotes the tumorigenicity of colorectal CSCs-enriched spheroid cells.^26, 31^

In human breast cancer, *KLF4* promoter demethylation and KLF4 protein expression indicate an unfavorable prognosis.^32, 33, 34^ KLF4 expression is positively correlated with tumor size, advanced grade and stage.^35^ We previously identified microRNAs, including microRNA-206 (miR-206) and miR-21, as direct transcriptional targets of KLF4 that promote RAS-extracellular signal-regulated kinase (ERK) signaling in triple-negative breast cancer (TNBC) cells.^36, 37^ Although on its own each miR exerts only subtle influences on RAS-ERK pathway activity, the coexpression of miR-206 and miR-21 potently represses the expression of pathway inhibitors including RASA1 and SPRED1. Furthermore, miR-206 directly represses KLF4 translation, constituting a feedback loop.^36^

In this study, we observed elevation of *KLF4* and miR-206 in the P^+^/E^+^ and ALDH^High^ MaCSC fractions. In TNBC cells, both KLF4 and miR-206 were critical for cell survival and *in vivo* tumor initiation. We identified the tumor-suppressor programmed cell death 4 (*PDCD4*) as a potential mediator of cell survival by miR-206. Furthermore, in TNBC cells we demonstrated the miR-206 regulation of a previously validated transcript, the gap junction protein connexin 43 (*CX43/GJA1*).^38^

Consistent with the elevated levels of miR-206 in MaCSCs, PDCD4 and CX43 levels were decreased. Supporting functional roles downstream of KLF4 and miR-206, suppression of either PDCD4 or CX43 led to anoikis resistance, an intrinsic property of CSCs.^7, 39, 40, 41, 42, 43^ Finally, further documenting a prosurvival role, miR-206 promoted chemoresistance of TNBC cells against paclitaxel or doxorubicin. Our studies identify KLF4 and miR-206 as functional MaCSC markers that mediate cell survival. Consequently, KLF4 and/or miR-206 may be therapeutically targeted to selectively cripple MaCSCs in TNBCs.

## Results

### miR-206 is highly expressed in basal-like breast cancers and MaCSCs

**Figure 1:**
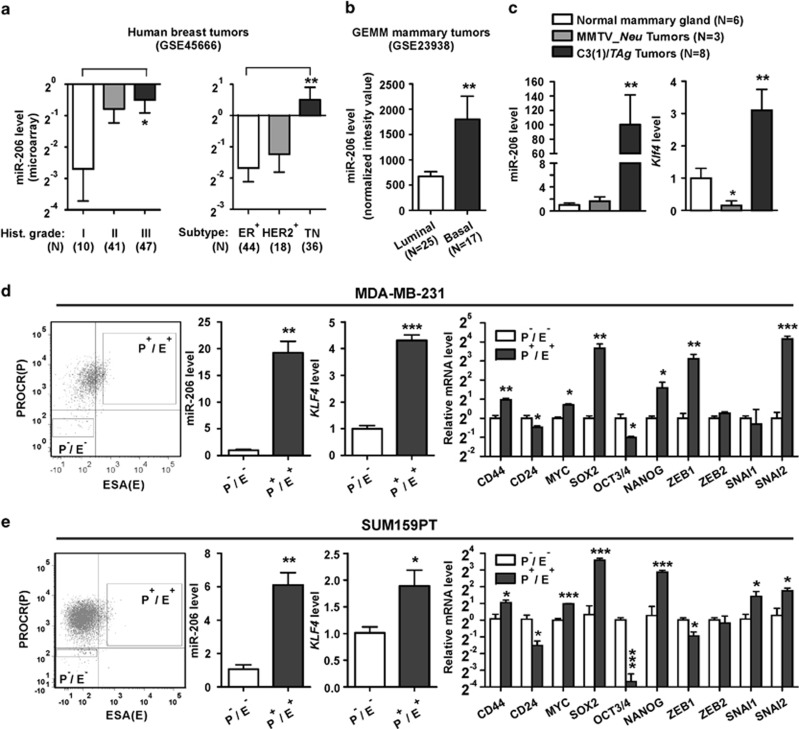
KLF4 and miR-206 are selectively expressed in basal-like mammary cancers and in the MaCSC population. (**a**) miR-206 levels were analyzed by microarray in 98 primary human breast tumors.^91^ The Gene Expression Omnibus (GEO) accession number is indicated. (*Columns*, mean; *bars*, s.e.m.; *Hist. grade*, histologic grade). (**b**) miR-206 levels were analyzed by microarray in 42 mammary tumors from GEMMs.^90^ The GEO accession number is indicated. (**c**) *Klf4* and miR-206 expression was evaluated in normal mammary tissues from FVB/N mice and in primary tumors arising in the MMTV-*Neu* and C3(1)/*TAg* GEMMs. RNA levels were determined by qRT–PCR. (**d**) MaCSCs were isolated from MDA-MB-231 cells by sorting using PROCR (P) and ESA (E) as described.^12^ Transcript levels were analyzed in P^+^/E^+^ and P^-^/E^-^ cells. (**e**) MaCSCs were isolated from SUM159PT cells and analyzed similarly as described above for MDA-MB-231 cells.^53^ For these cells, the P^+^ profile was used as a surrogate for CD44^+^/CD24^-^.^10^ (*P<0.05; **P<0.01; ***P<0.001).

KLF4 protein levels correlate with an aggressive phenotype in breast tumors.^32, 33, 35^ Similar to KLF4, miR-206 was increased in human tumors of advanced histological grade (Figure 1a, left panel). Consistent with studies that identified upregulation of miR-206 in ER^-^ breast tumors, miR-206 levels were elevated in TNBCs compared with both ER^+^ and HER2^+^ human subgroups (Figure 1a, right panel).^44, 45^ Enrichment of miR-206 was similarly observed in murine basal-like mammary tumors (Figure 1b). Compared with normal mammary tissues or tumors arising in the luminal MMTV_*Neu* model,^46^ we observed upregulation of both *KLF4* and miR-206 in basal-like tumors derived from the C3(1)/*SV40 large T antigen* (C3(1)/*TAg*) genetically engineered mouse model (GEMM) (Figure 1c). These results are consistent with the direct regulation of miR-206 by KLF4 as previously reported.^37^

MaCSCs are enriched in the triple-negative subgroup of breast cancer and are thought to contribute to the aggressive behavior of these cancers.^47, 48, 49^ Similar to human and murine mammary carcinoma cells displaying high ALDH activity,^11, 50, 51, 52^ MDA-MB-231 TNBC cells displaying the P^+^/E^+^ surface marker profile represent TICs.^12^ For SUM159PT cells, CD44^+^/CD24^-^/ESA^+^ subset was previously identified as TICs.^53^ As the P^+^ phenotype is a surrogate for the CD44^+^/CD24^-^ profile, the P^+^/E^+^ SUM159PT cells are likely to represent MaCSCs.^10^

We analyzed *KLF4* and miR-206 levels in flow-sorted sub-populations of MDA-MB-231 cells (Figure 1d, left panel). Compared with non-MaCSCs (that is, P^-^/E^-^), miR-206 and *KLF4* were increased in the P^+^/E^+^ sub-population (Figure 1d, middle panels). Using P^+^/E^+^ cells, we profiled the expression of other genes associated with stem-like cell phenotypes.^9, 14, 18, 19^ Compared with P^-^/E^-^ cells, the expression of *CD44, MYC, SOX2, NANOG, ZEB1* and *SNAI2* was upregulated in P^+^/E^+^ cells, whereas *CD24* and *POU5F1* (*OCT3/4*) expression were decreased (Figure 1d, right panel). Similarly, the P^+^/E^+^ fraction of SUM159PT cells displayed elevated levels of *KLF4* and miR-206, and showed a similar stem cell marker profile as the MDA-MB-231 cells (Figure 1e). These results associate *KLF4* and miR-206 with the MaCSC phenotype in human breast cancer models.

### *KLF4* and miR-206 are enriched in MaCSCs derived from human patient-derived xenografts (PDXs) and the C3(1)/*TAg* GEMM

**Figure 2:**
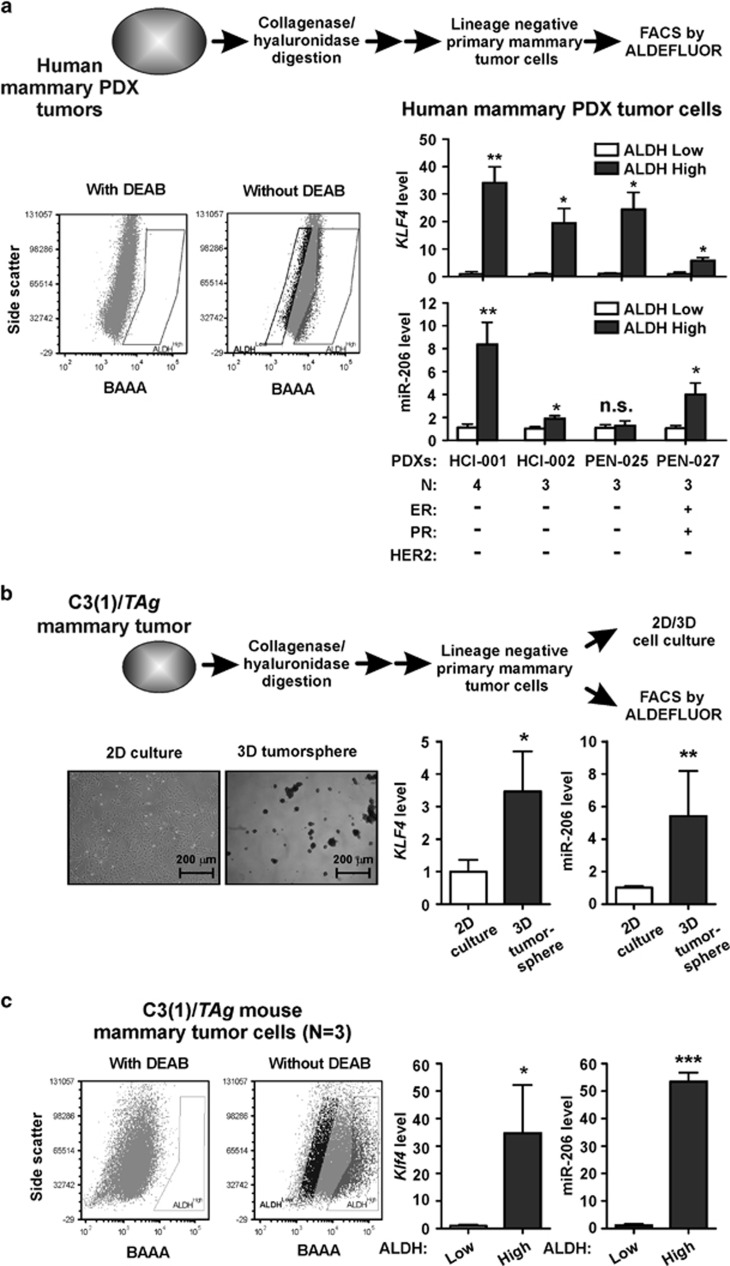
*KLF4* and miR-206 are enriched in ALDH^High^ MaCSCs derived from human PDXs and the C3(1)/*TAg* GEMM. (**a**) *KLF4* and miR-206 levels were measured in MaCSCs purified in replicate manner from four cases of human mammary carcinoma passaged as xenografts in mice (PDXs). Purified lineage-negative (Lin^-^) cells were sorted based on ALDH activity. Fluorescence was analyzed in the presence of the ALDH substrate BAAA and in presence/absence of the ALDH inhibitor DEAB. The number of xenografted tumors that were analyzed for each case is indicated below the column data (*bars*, s.e.m.). n.s., not significant. (**b**) Lin^-^ cells were isolated from the spontaneous mammary tumors arising in C3(1)/*TAg* females (*N*=3). Cells from each animal were grown as either adherent monolayers (2D) or in suspension (3D) for 7–10 days. Photomicrographs (left) depict the morphology of cultured cells. RNA was extracted from 2D or 3D cell cultures and *Klf4* and miR-206 levels were determined. (**c**) *Klf4* and miR-206 levels were measured in the ALDH^High^ tumor cells isolated from C3(1)/*TAg* animals (*N*=3, see panel **b**). ALDH^High^ tumor cells derived from other GEMMs of mammary cancer have been demonstrated to be enriched for TICs^50, 52^ (*P<0.05; **P<0.01; ***P<0.001).

*KLF4* was similarly consistently elevated in lineage-negative (Lin^-^)/ALDH^High^ MaCSCs isolated from human mammary tumor tissues that were passaged as PDXs (Figure 2a). miR-206 was upregulated in three of these four cases. Notably, none of these tumors displayed an appreciable CD44^+^/CD24^-^ MaCSC population (data not shown), consistent with the variable expression of these markers in patient samples.^10, 54, 55^

Tumorspheres are enriched for MaCSCs.^7, 8^ Compared with cells grown in adherent (two-dimensional (2D)) monolayers, tumorspheres formed from the Lin^-^ cells of C3(1)/*TAg* mammary tumors showed elevated levels of *Klf4* and miR-206 (Figure 2b). ALDH^High^ cells from other mammary cancer GEMMs were previously shown to have properties of MaCSCs.^50, 52^ Similar to the human tumors, Lin^-^/ALDH^High^ cells of C3(1)/*TAg* mammary tumors also had increased *Klf4* and miR-206 relative to ALDH^Low^ cells (Figure 2c). These results identify *KLF4* and miR-206 as MaCSC markers and potential mediators of MaCSC malignant properties.

### KLF4 and miR-206 can promote MaCSC abundance

**Figure 3:**
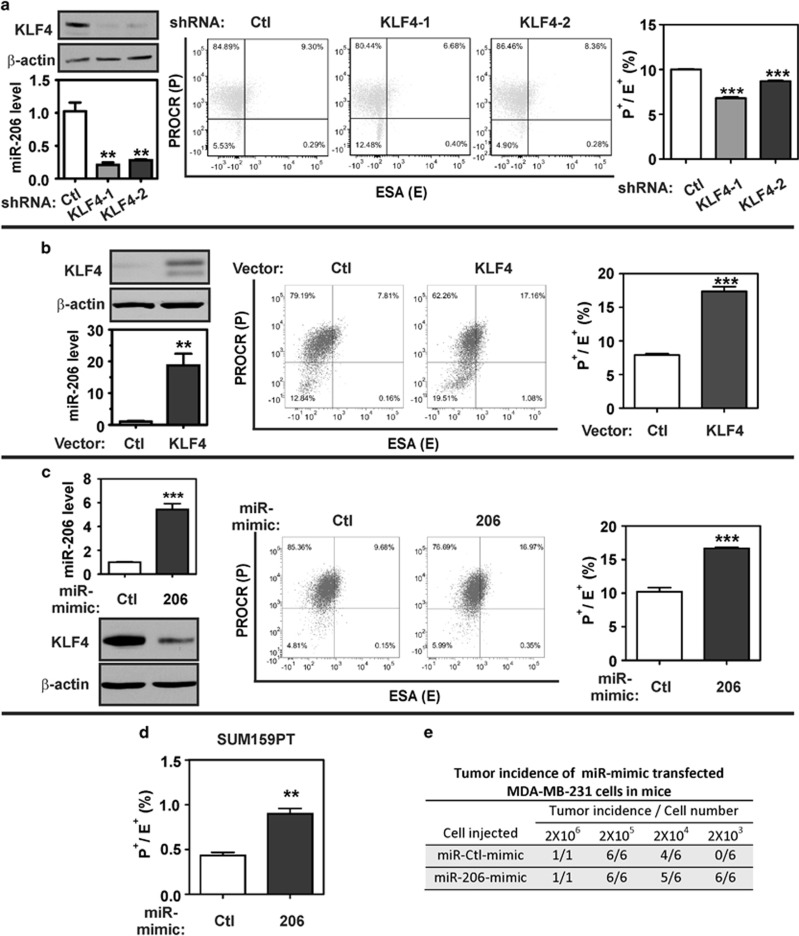
KLF4 and miR-206 promote MaCSC abundance. (**a**) MDA-MB-231 cells were transduced with lentiviral vectors expressing KLF4 short hairpin RNAs or a non-targeting control (Ctl). KLF4 protein expression was analyzed by immunoblot (left upper panel). β-Actin served as a loading control. miR-206 levels were measured by stem loop qRT–PCR (left lower panel). The cell surface marker profile of the transduced cells was analyzed by flow cytometry (representative scatter plot, middle panel; column data, right panel) (*N*=3; *bars*, s.e.m.). (**b**) MDA-MB-231 cells were transduced with a retroviral vector encoding KLF4 or empty vector (Ctl). KLF4 and miR-206 levels were analyzed in these cells (left panels) and the MaCSC abundance was determined by flow cytometry. (**c**) MDA-MB-231 cells were transfected with either miR-206 mimic or control oligonucleotides (Ctl) and then analyzed as in the previous panels. (**d**) SUM159PT cells were transfected with the indicated miR-mimics and then analyzed as in the previous panels. (**e**) MDA-MB-231 cells were transfected with miR-206 mimic or control. The indicated number of cells were mixed with matrigel (50% (vol/vol) in DMEM) and injected into NSG mice. Tumor initiation was measured at 4 weeks post-injection (**P<0.01; ***P<0.001).

To determine the effect of KLF4-miR-206 signaling on MaCSC abundance, we depleted KLF4 in MDA-MB-231 cells using two distinct lentiviral short hairpin RNA constructs (Figure 3a, left upper panel). Consistent with previous studies, miR-206 was suppressed following KLF4 knockdown (Figure 3a, left lower panel). In addition, P^+^/E^+^ cell abundance was modestly decreased upon KLF4 depletion (Figure 3a, middle and right panels). Conversely, gain-of-function experiments showed that exogenous KLF4 promoted both miR-206 levels and the abundance of P^+^/E^+^ cells (Figure 3b).

We next sought to determine whether miR-206 could have a causal role downstream of KLF4 to regulate MaCSC abundance. As expected, transfection of miR-206 mimic into MDA-MB-231 cells elevated the miR-206 level as detected by quantitative reverse transcription and PCR (qRT–PCR; Figure 3c, left upper panel). In addition, the level of KLF4 was suppressed, attributed to direct regulation of KLF4 protein translation by miR-206 (Figure 3c, left lower panel).^36^ Despite the reduced levels of KLF4, miR-206-transfected cells displayed higher P^+^/E^+^ cell abundance relative to the control cells (Figure 3c, right panel). Similar regulation of P^+^/E^+^ cell abundance by miR-206 was observed for SUM159PT cells (Figure 3d). These results establish miR-206 as a potential effector of KLF4 for regulation of MaCSC abundance.

To determine whether miR-206 can promote the MaCSC phenotype, we assayed by limiting dilution the capability of miR-206-transfected MDA-MB-231 cells to initiate tumors *in vivo*. Consistent with an increased number of P^+^/E^+^ cells, miR-206-transfected cells formed tumors more efficiently in NOD/SCID-gamma (NSG) mice compared with control cells (Figure 3e; 2 × 10^3^ cells, *P*=0.0022). These results implicate miR-206 as an effector of KLF4 that promotes tumor initiation.

### Endogenous KLF4 and miR-206 promote tumor cell survival and *in vivo* tumorigenesis

**Figure 4:**
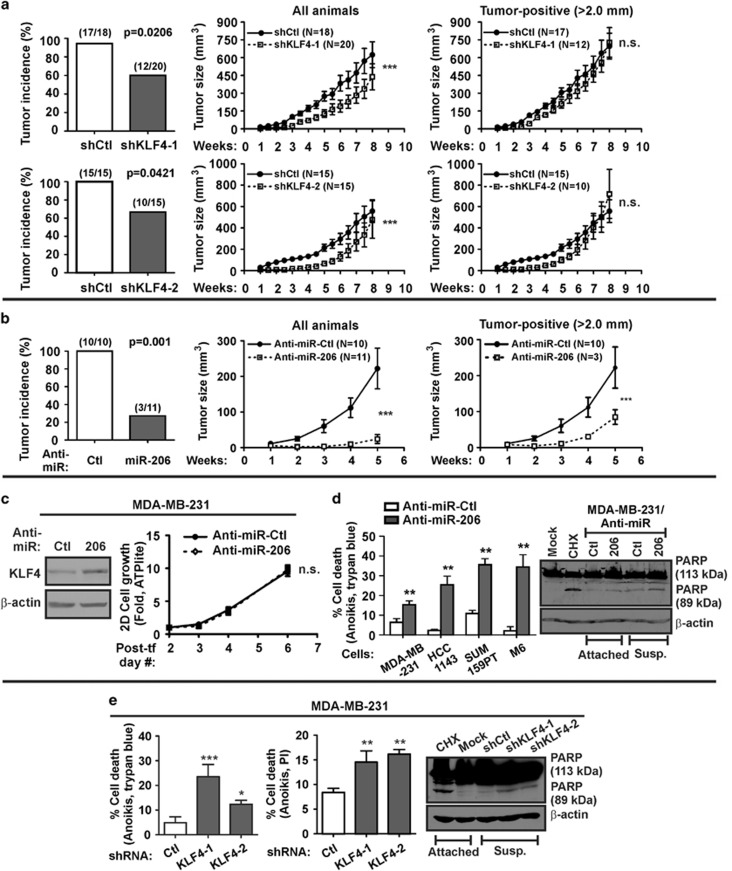
Endogenous KLF4-miR-206 signaling promotes *in vivo* tumorigenesis and cell survival. (**a**) KLF4-depleted and control MDA-MB-231 cells were orthotopically injected into athymic nude mice. Tumor initiation and tumor size were determined twice per week using digital calipers (right panels; *bars*, s.e.m.). (**b**) MDA-MB-231 cells were transfected with the indicated anti-miRs. Briefly, cells were subjected to sequential transfections *in vitro*. At 2 days post-transfection, the cells were injected into athymic nude mice. Tumor incidence and growth were measured as described above. (**c**) Residual transfected cells (see panel **b**) were directly lysed for immunobot analysis (left panel) or else placed in culture for 2D cell proliferation analysis (right panel, ATPlite; *N*=6, *bars*, s.d.). *Post-tf*, post-transfection. (**d**) TNBC cells were transfected with either anti-miR-206 or anti-miR-Ctl and then deprived of matrix for 24 h. Anoikis was measured by Trypan blue exclusion (left panel, *N*=3, *bars*, s.d.). In parallel, cells were assayed by immunoblot analysis of cleaved PARP. Cyclohexamide (CHX) treatment served as a positive control for induction of cell death. (**e**) Anoikis was measured in KLF4-depleted MDA-MB-231 cells or control cells by Trypan blue exclusion (*N*=3, *bars*, s.d.), by flow cytometric analysis of propidium iodide (PI)-stained cells (*N*=3, *bars*, s.d.), and by analysis of cleaved PARP. n.s., not significant (*P<0.05; **P<0.01; ***P<0.001).

We next examined the impact of endogenous KLF4-miR-206 signaling on tumor initiation. Depletion of KLF4 reduced the tumor initiation rate of MDA-MB-231 cells in athymic nude mice (Figure 4a, left panels). This decrease in tumor incidence was reflected by the reduced mean tumor volume for all animals combined (Figure 4a, middle panels). Indicating that the major effect of KLF4 in this setting is restricted to tumor initiation, analysis of the tumor-positive subset revealed little difference in the tumor growth rate between KLF4-depleted cells and the control (Figure 4a, right panels).

To study the role of endogenous miR-206 during *in vivo* tumorigenesis, we analyzed the tumorigenicity of MDA-MB-231 cells treated by *in vitro* transfection of anti-sense oligonucleotides (anti-miR-206). Compared with cells transfected with the control, anti-miR-206 treatment reduced both tumor incidence and tumor growth (Figure 4b). As an indicator of successful transfection, KLF4 expression was increased (Figure 4c, left panel). Effects on tumor growth were not likely attributed to differences in cell proliferation rates, as anti-miR-206 had little effect (Figure 4c, right panel).

The critical role of endogenous miR-206 for tumor initiation following orthotopic injection, despite its minimal effects on cell proliferation or MaCSC abundance, pointed to a potential role in regulating cell survival. We therefore assayed for resistance to cell death following matrix deprivation (anoikis), an intrinsic property of CSCs.^7, 39, 40, 41, 42, 43^ Indeed, consistent with our previous report that analyzed two human TNBC cell lines,^37^ anti-miR-206 transfection sensitized several human TNBC models and a murine basal-like mammary cancer model (that is, M6 cells) to anoikis (Figure 4d, left panel). Consistent results were obtained when anoikis was analyzed by poly ADP ribose polymerase (PARP) cleavage (Figure 4d, right panel). In support of a prosurvival role for endogenous miR-206, depletion of KLF4 sensitized TNBC cells to anoikis (Figure 4e). These results suggest that endogenous KLF4 exerts a prosurvival effect by induction of miR-206.

### miR-206 suppresses the translation of the tumor-suppressor *PDCD4* and promotes tumor cell survival

We previously reported that RAS-ERK signaling, a prosurvival pathway, is maintained in TNBC cells by KLF4, in part through its regulation of miR-206.^37^ In contrast to the prominent effect of miR-206 on tumor initiation and cell survival, on its own this miR has only limited effects on ERK activity.^37^ We therefore sought to better understand how endogenous miR-206 can promote anoikis resistance.

The tumor-suppressor *PDCD4* was identified as a potential miR-206 targeted transcript by multiple miR-target prediction tools.^37, 56^ PDCD4 is a negative regulator of RAS-ERK-AP1 signaling and protein translation, and promotes breast cancer cell apoptosis.^57, 58, 59^ We therefore analyzed *PDCD4* as a miR-206-regulated transcript.

**Figure 5:**
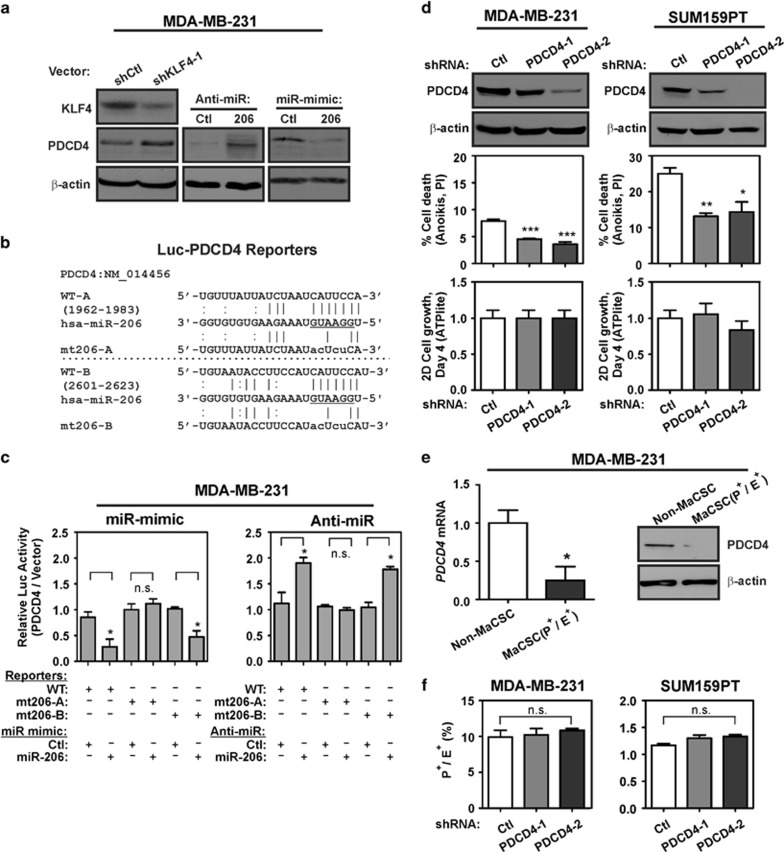
miR-206 suppresses the translation of the tumor-suppressor *PDCD4*. (**a**) PDCD4 levels were determined by immunoblot analysis of the indicated cells. (**b**) Alignment of the *PDCD4* 3′UTR region indicating two potential miR-206 binding sites, WT-A and WT-B. The miR-206 seed sequence is underlined. Mutated miR-206 binding sites in the *PDCD4* 3′ UTR that were utilized in translational reporter assays are indicated (mt206-A and mt206-B). (**c**) For analysis of PDCD4 protein translation, MDA-MB-231 cells were co-transfected with reporters in combination with either miR-mimic (left panel) or anti-miR (right panel). The normalized activity of the reporters relative to empty luc vector was analyzed 24 h post-transfection (*N*=3; *bars*, s.e.m.). (**d**) PDCD4 was depleted in the indicated TNBC cells and PDCD4 levels were determined by immunoblot (upper panels). Cells were suspended in 3D culture for 24 h, and anoikis was measured by flow cytometric analysis of propidium iodide (PI)-stained cells (middle panels; *N*=3; *bars*, s.e.m.). Following 4 days of 2D culture, the relative cell number of PDCD4-depleted cells and control cells was determined by the ATPlite assay (*N*=6; *bars*, s.d.). (**e**) PDCD4 mRNA and protein expression was analyzed in the indicated sub-populations of TNBC cells. Non-MaCSCs comprises the P^+^/E^-^ and P^-^/E^-^ subgroups. The immunoblot data correspond to one of the three independent experiments that analyzed mRNA levels (*N*=3, *bars*, s.e.m.). (**f**) MaCSC abundance was analyzed in PDCD4-depleted TNBC cells and control cells (*N*=3; *bars*, s.d.). n.s., not significant (*P<0.05; **P<0.01; ***P<0.001).

Consistent with regulation of *PDCD4* by miR-206, KLF4 depletion in MDA-MB-231 cells increased PDCD4 expression (Figure 5a, left panel). Similarly, although anti-miR-206 treatment elevated PDCD4, transfection of miR-206 mimic was suppressive (Figure 5a, middle and right panels). Direct regulation of PDCD4 by miR-206 was determined using translational reporter assays. Fragments of the *PDCD4* 3′ UTR containing two putative miR-206-binding sites (denoted WT-A and WT-B; Figure 5b) were cloned downstream of the open reading frame of firefly luciferase (luc). Relative to the controls, in MDA-MB-231 cells miR-206 mimic repressed WT-reporter luc activity by 72%, and anti-miR-206 induced the reporter by 1.9-fold (Figure 5c). Reporter regulation by miR-206 was abolished by mutation of site WT-A, but not by mutation of site WT-B, thus identifying site WT-A as a functional miR-206-binding site (Figure 5b and c). In agreement with previous studies, PDCD4 depletion in TNBC cells promoted resistance to anoikis, with little or no effect on 2D proliferation (Figure 5d).

Consistent with miR-206 regulation of *PDCD4* in MaCSCs, the P^+^/E^+^ sub-population of MDA-MB-231 cells exhibited decreased levels of PDCD4 mRNA and protein compared with non-MaCSCs (Figure 5e). In TNBC cells, the depletion of PDCD4 was not sufficient to alter the abundance of the P^+^/E^+^ fraction (Figure 5f). These results appear to support a selective role of PDCD4 for suppression of tumor cell survival.

### miR-206 promotes cell survival by suppressing *CX43* in MaCSCs

Our identification of miR-206 regulation of *PDCD4* led us to seek additional targets of this miR that may be important for promoting cell survival. DIANA-miRPath analysis identifies gap junction signaling as the top-ranked miR-206-regulated pathway (*P*=2.58 × 10^−6^).^60^ Among the targeted gap junction proteins, CX43 is a validated miR-206-regulated transcript, as previously shown in muscle cells.^38, 61^ CX43 is deficient in human breast tumor cells and MaCSCs, and may exert a tumor-suppressor role.^62, 63, 64, 65, 66, 67, 68^

**Figure 6:**
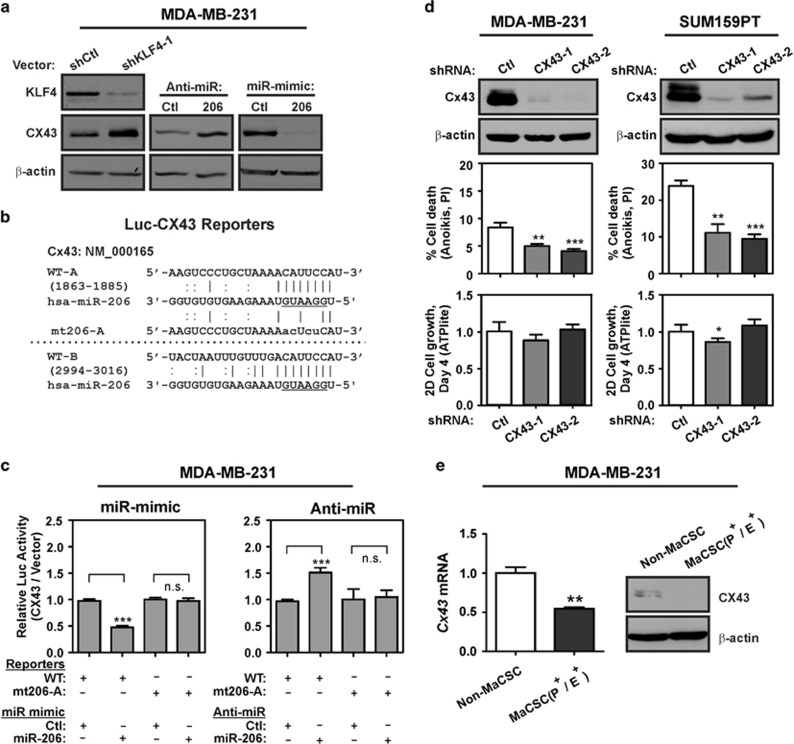
KLF4-miR-206 signaling suppresses CX43 in MaCSCs. (**a**) CX43 expression was analyzed in shKLF4 cells and control cells by immunoblot. Similarly, CX43 expression was analyzed in cells transfected with the indicated miR mimic or anti-miR. (**b**) Alignment of *CX43* 3'UTR region, indicating two previously validated miR-206 binding sites, WT-A and WT-B.^38^ The miR-206 seed sequence is underlined. The mutation generated in miR-206 binding site A is indicated (mt206-A). (**c**) For analysis of CX43 protein translation, MDA-MB-231 cells were co-transfected with reporters in combination with anti-miR (left panel) or miR-mimic (right panel). The normalized activity of the reporters relative to empty luc vector was analyzed at 24 h post-transfection. (**d**) CX43 expression was assayed in CX43-depleted TNBC cells and control cells (upper panels). Cells were suspended in 3D culture for 24 h, and anoikis was measured by flow cytometric analysis of propidium iodide (PI)-stained cells (middle panels, *N*=3; *bars*, s.e.m.). Following 4 days of 2D culture, the relative cell number of CX43-depleted cells and control cells was determined by the ATPlite assay (lower panels, *N*=6; *bars*, s.d.). (**e**) CX43 mRNA and protein expression was analyzed in the indicated sub-populations of MDA-MB-231 cells (*N*=3; *bars*, s.e.m.). Non-MaCSCs were composed of the P^+^/E^-^ and P^-^/E^-^ subgroups. n.s., not significant (*P<0.05; **P<0.01; ***P<0.001).

Consistent with its regulation by miR-206 in breast cancer cells, CX43 was increased in KLF4-depleted MDA-MB-231 cells (Figure 6a, left panel). Similarly, inhibition of miR-206 led to elevated CX43 levels, and transfection of miR-206 mimic was suppressive (Figure 6a, middle and right panels). In TNBC cells, the activity of a translational reporter containing the *CX43* 3' UTR was induced by 1.5-fold following anti-miR-206 treatment, and suppressed by 53% following transfection of miR-206 mimic (Figures 6b and c). Supporting the direct regulation of CX43 by miR-206 in breast tumor cells, mutation of site A (mut206-A) abolished regulation by miR-206 (Figure 6c). Similar to PDCD4 depletion, suppression of CX43 in TNBC cells promoted resistance to anoikis, with only subtle effects on cell proliferation (Figure 6d).

Compared with the non-MaCSC fraction, P^+^/E^+^ MDA-MB-231 cells displayed lower CX43 mRNA and protein (Figure 6e). These results support a previous study that reported low CX43 expression in mammary TICs.^64^ Similarly to PDCD4, knockdown of CX43 did not consistently alter the P^+^/E^+^ cell abundance in TNBC cells, suggesting a selective role in tumor cell survival (data not shown).

### miR-206 confers chemoresistance in TNBC cells

**Figure 7:**
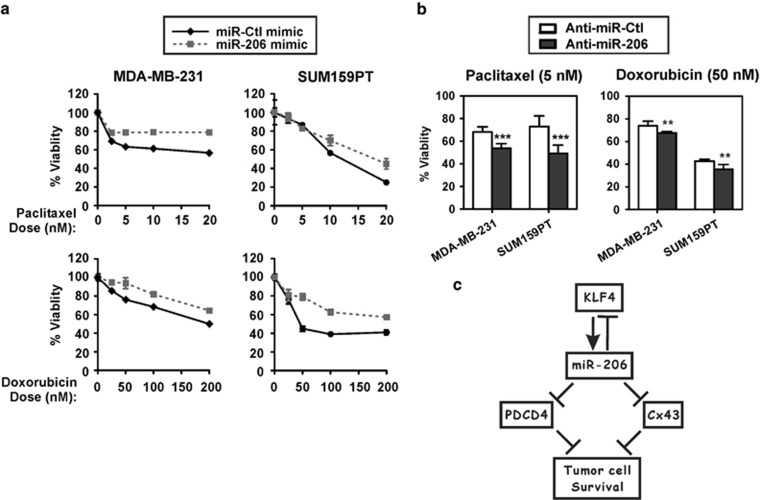
miR-206 promotes chemoresistance in TNBC cells. (**a,** **b**) TNBC cells were transfected as indicated. At 48 h post-transfection, cells were treated with the indicated concentrations of paclitaxel or doxorubicin for a duration of 72 h. Cell viability was determined by the ATPlite assay (*N*=6; *bars*, s.d.). (**c**) Schematic of KLF4-miR-206 regulation of *PDCD4* and *CX43.* (**P<0.01; ***P<0.001).

Consistent with the promotion of cell survival by miR-206 as determined by anoikis assays, TNBC cells transfected with miR-206 mimic were more resistant to paclitaxel or doxorubicin (Figure 7a). Furthermore, inhibition of the endogenous miR-206 moderately sensitized TNBC cells to either agent (Figure 7b). Collectively, these results link pluripotency factor signaling and the enhanced cell survival of MaCSCs, supporting roles of KLF4-miR-206 signaling for breast tumor cell survival, chemoresistance, and tumor initiation through the repression of *PDCD4* and *CX43* (Figure 7c).

## Discussion

CSCs were first identified in hematopoietic malignancies and subsequently in solid tumors such as breast cancer.^1, 3, 5, 69^ Despite substantial progress, questions remain regarding the relationship of CSCs to the adult stem cells of normal tissue, and the nature of the signaling pathways that regulate CSC properties.^6^ Despite this uncertainty, it is clear that CSCs represent a highly malignant sub-population of tumor cells with the capability to resist therapy.^3, 5^

In TNBC cells, KLF4 directly regulates miR-206 transcription, and depletion of KLF4 consistently results in loss of the vast majority of miR-206.^36, 37^ In this study, we identified KLF4 and miR-206 as critical promoters of breast tumor cell survival. Both factors were preferentially expressed in the MaCSCs purified from 2D cell culture models of TNBC, from tumorspheres cultured in 3D, from human PDXs and from primary mouse mammary cancers. As shown by anti-miR treatment of TNBC cells, endogenous miR-206 directly repressed the translation of the tumor suppressors *PDCD4* and *CX43* and promoted tumor cell survival, chemoresistance and *in vivo* tumor initiation. Immunoblot analysis of MaCSCs revealed suppressed levels of both PDCD4 and CX43. Mirroring the role of endogenous miR-206, depletion of each tumor suppressor did not alter the abundance of CSCs, but instead enhanced tumor cell survival consistent with previous reports.^57, 70^

miRs can act as critical factors for regulating the abundance and/or survival of MaCSCs.^71, 72, 73, 74^ In skeletal muscle, miR-206 is important for repression of PAX7 during stem cell differentiation, and for muscle regeneration following injury.^75, 76, 77, 78, 79^ In a mammary cancer context, miR-206 expression is elevated in ER^-^ tumors, which are enriched for MaCSCs.^44, 45, 47, 48, 49^ In agreement with previous studies, we observed that miR-206 is upregulated in human breast cancers that display a higher grade, in human TNBCs and in basal-like mammary tumors derived from the C3(1)/*TAg* GEMM (Figure 1a and c).

Multiple previous studies have reported that enforced expression of miR-206 can suppress tumor cell proliferation, invasion or metastasis.^45, 80, 81, 82, 83^ These tumor-suppressor-like effects of miR-206 may result from higher level enforced expression of the exogenous miR. In this study, suppression of endogenous miR-206 blocked tumor initiation, and moderate (fivefold) overexpression of exogenous miR-206 promoted initiation in a limiting dilution assay. In addition, we observed that either exogenous or endogenous miR-206 could promote malignant properties including tumor cell survival and drug resistance.

Depletion of endogenous KLF4 suppressed *in vivo* tumor initiation by MDA-MB-231 cells in athymic nude mice, yet had little effect on the growth rate of established tumors. Similarly as observed for KLF4, transient inhibition of endogenous miR-206 by anti-miR-206 transfection suppressed tumor initiation *in vivo* but did not alter the *in vitro* proliferation or the MaCSC abundance. These results suggest that endogenous KLF4 can signal through miR-206 to promote tumor initiation, probably by impacting cell survival rather than MaCSC abundance. In contrast, exogenous KLF4 or miR-206 promoted MaCSC abundance, mirroring the role of exogenous KLF4 for generation of induced pluripotent stem cells.^18, 19^ It will be interesting to determine whether miR-206 similarly influences the generation of induced pluripotent stem cells.

In this study, we have identified endogenous KLF4 and a downstream effector, miR-206, as functional markers and prosurvival factors that are enriched in MaCSCs. Prosurvival signaling by miR-206 was attributed to direct regulation of *PDCD4* and *CX43,* and miR-206 enhanced the chemoresistance of TNBC cells. Our study, therefore, provides a rationale for miR-206-directed antago-miR therapy for the sensitization of the MaCSCs.^74, 84, 85, 86, 87, 88^

## Materials and methods

### Cell lines, cell culture and drug treatments

MDA-MB-231 cells were provided by Katri S Selander (University of Alabama at Birmingham, AL, USA), SUM159PT cells were provided by Gary L Johnson (University of North Carolina at Chapel Hill, NC, USA) and M6 mammary carcinoma cells derived from the C3(1)/SV40 *TAg* mouse model were provided by Jeffrey E Green (NIH). HCC1143 cells were from ATCC (Manassas, VA, USA). Cells were maintained as subconfluent monolayers as previously described.^36, 37^

For chemoresistance experiments, cells were treated with the indicated doses of paclitaxel (Sigma, St Louis, MO, USA) or doxorubicin (Merck, Billerica, MA, USA) for 72 h. Cells were treated with cycloheximide (Sigma) at 20 μg/ml for 24 h. Cell proliferation was determined using the ATPlite Luminescence Assay System (PerkinElmer, Waltham, MA, USA).

### Retroviral transduction

Suppression studies utilized the following pGIPZ lentiviral shRNAmir plasmids (V2LHS_28277 – shKLF4-1, V3LHS_410934 – shKLF4-2, V3LHS_411731 – shCX43-1, V3LHS_411733 – shCX43-2, V3LHS_366084 – shPDCD4-1, V3LHS_366087 – shPDCD4-2; GE Dharmacon/Open Biosystems, Lafayette, CO, USA). The retroviral vector pBABEpuro-HA-KLF4 and viral transduction was previously described.^36^ Cells were selected using puromycin (1 μg/ml).

### Plasmid construction

pMIR-REPORT firefly luciferase vector was purchased from Ambion (Austin, TX, USA). pRL-TK *Renilla* luc reporter was obtained from Promega (Madison, WI, USA).

Complementary DNA clones containing fragments of the 3′ UTR of *PDCD4* (clone ID: NM_014456) and *CX43/GJA1* were purchased from Open Biosystems and OriGene (Rockville, MD, USA), respectively.

To construct a WT *PDCD4* translational reporter, a 1.7-kb fragment representing the 3′ UTR was excised using *Mlu*I and inserted into *Mlu*I-digested pMIR-REPORT. To construct a WT *CX43/GJA1* translational reporter, a 1.7-kb fragment representing the *CX43* 3′ UTR was generated by sequential treatment with *Eco*RI, Klenow fragment and *Mlu*I. This fragment was inserted into pMIR-REPORT vector that was prepared by sequential treatment with *Sac*I, Klenow fragment and *Mlu*I.

*PDCD4* and *CX43* reporters with mutation in the miR-seed complementary regions were generated by PCR mutagenesis. Oligonucleotides are listed in Supplementary Table S1. WT reporters were mutated so as to conserve the predicted secondary structure of the 3′ UTR.^89^ Cloned PCR products were confirmed by sequence analysis.

### Transient transfection and translation reporter assays

The following anti-miR inhibitors (AM) and miR-mimics (PM) were obtained from Ambion and diluted to 20 μM in nuclease-free water: hsa-miR-206 (AM10409, PM10409), AM-negative control (AM17010), and PM-negative control (AM17110). Cells were subjected to reverse transfection and, 24 h later, forward transfection was performed as described.^36^ At 24 h after the start of the forward transfection, cell extracts were prepared for expression studies, or cells were used for phenotypic studies. Translational reporter assays were performed following just one transfection, at 24 h after the start of the reverse transfection. Inhibitors/mimics were co-transfected with reporter plasmids, and Dual-Luciferase Reporter Assays (Promega) were performed as described.^36^

### Immunoblot analysis and antibodies

Cell extracts for immunoblot analysis were prepared as previously described.^36^ PARP cleavage assays were performed as recommended (Roche, Indianapolis, IN, USA). Following electrophoresis, proteins were transferred onto nitrocellulose membranes and probed with the indicated antibody: KLF4 (Santa Cruz Biotechnology, Dallas, TX, USA), PDCD4 (Rockland Immunochemicals, Philadelphia, PA, USA), CX43 (Sigma), PARP (Roche) or β-actin (Santa Cruz Biotechnology). Bound antibodies were detected using Pierce ECL Western Blotting Substrate (ThermoFisher Scientific, Waltham, MA).

### Animal studies

Female athymic nude mice (Crl:NU(NCr)-Foxn1^nu^, Charles River, Frederick, MD, USA) were obtained at 6–8 weeks of age. In all, 2 × 10^6^ cells were suspended in Dulbecco's modified Eagle's medium (DMEM) and injected into the fourth mammary fat pad. For tumor initiation/limiting dilution assays, NSG (NOD.Cg-Prkdcscid Il2rgtm1Wjl/SzJ; Jackson Lab, Bar Harbor, ME, USA) were obtained at 6–8 weeks of age. Tumor cells were suspended in DMEM containing matrigel (50% (vol/vol)) and injected into the fourth mammary fat pad. Tumors were measured semiweekly using digital calipers. Tumor volume was determined by π(L1 × L2^2^)/6 (L1, long axis; L2, short axis), and tumor initiation was defined as ⩾2 mm for both L_1_ and L_2_. Animal procedures were performed under an approved protocol.

### Isolation of mammary carcinoma cells from tumors

Human mammary cancer tissue was passaged as PDXs in NSG mice. HCI-001 and HCI-002 were obtained from Alana L Welm, University of Utah, and PEN-025 and PEN-027 were obtained from the West Virginia University Tissue Bank. PDX tumors and tumors arising in female C3(1)/*TAg* mice were harvested upon reaching a size of 1-2 cm^3^. To isolate mammary carcinoma cells, tumors were minced and suspended in DMEM/F12 containing Gentle Collagenase/Hyaluronidase (STEMCELL Technologies, Vancouver, BC, Canada) and then processed as recommended by the manufacturer. Briefly, tumor cell suspensions were incubated with mild agitation at 37 °C for 15 h. Red blood cells were lysed by the addition of 0.16 M Tris-NH_4_Cl (pH 7.6) and incubation at 25 °C for 3 min. Red blood cell lysis was stopped by the addition of DMEM/F12 containing 10% fetal bovine serum . The suspension was centrifuged and the resulting cell pellet was washed twice with DMEM/F12 containing 10% fetal bovine serum , resuspended in Trypsin-EDTA (0.25% Media Tech, Corning, NY, USA) for 3 min with disaggregation by pipette, and then washed once again. Cells were resuspended in dispase and DNAse I (STEMCELL Technologies) at final concentrations of 4.2 mg/ml and 192 μg/ml, respectively. The cells were centrifuged and the cell pellet was resuspended in Hank's balanced salt solution containing 10 mM HEPES-KOH, pH 7.2 and 2% fetal bovine serum .

Depletion of lineage-positive (Lin^+^) cells from prepared tumor cell suspensions was performed using an AutoMACS sorter (Miltenyi Biotec, San Diego, CA, USA). Briefly, cells were suspended in ice cold staining buffer (phosphate-buffered saline supplemented with 0.5% (wt/vol) bovine serum albumin) and blocked with 10 μg/ml mouse immunoglobulin G (Sigma) for 15 min. Cells were stained with the following biotin-conjugated antibodies (BD Bioscience, San Jose, CA, USA): anti-mouse-CD31 (clone 390), anti-CD45 (clone 30-F11), anti-TER-119 (clone TER-119). Anti-CD140b was from eBioscience, San Diego, CA, USA (clone APB5). Cells were washed with labeling buffer (phosphate-buffered saline, pH 7.2 containing 0.5% bovine serum albumin and 2 mM EDTA) and incubated with streptavidin microbeads (Miltenyi Biotec) before magnetic cell sorting as recommend by manufacturer.

### Analysis and purification of MaCSCs

For analysis of PROCR/ESA expression, cells were blocked with 10 μg/ml normal human immunoglobulin G (R&D System, Minneapolis, MN, USA) in ice cold staining buffer (phosphate-buffered saline supplemented with 1% (vol/vol) fetal bovine serum) for 15 min. Cells were stained with anti-human PROCR-APC (clone RCR-227; eBioscience) and anti-human ESA-PerCP-Cy5.5 (clone EBA-1; BD Bioscience). Cells were centrifuged at 300 × *g* for 5 min at 4 °C, and washed twice with staining buffer before analysis.

ALDH activity was evaluated by flow cytometry using the ALDEFLUOR assay (STEMCELL Technologies). Cell sorting or flow cytometry was performed on a BD FACSAria using BDFACSDiva software version 6.1, or on a BD Fortessa using BDFACSDiva software version 7.0 (Becton Dickinson, San Jose, CA, USA). For analysis, a minimum of 10 000 events were collected for each sample. The data were analyzed by using FCS Express 4 Research Edition software (*De Novo* software, Glendale, CA, USA).

### Tumorsphere formation and anoikis assays

To grow tumorspheres, 2 × 10^4^ Lin^-^ cells were placed in suspension cultures in low attachment plates (Costar, Corning, NY, USA) using DMEM/F12 supplemented with B27, 4 μg/ml heparin, 20 ng/ml epidermal growth factor, 20 ng/ml fibroblast growth factor and 1% (wt/vol) methylcellulose. For analysis of anoikis, cells were suspended in culture as previously described.^37^ Cell death was analyzed by propidium iodide staining and flow cytometry (Invitrogen, Carlsbad, CA, USA), by Trypan blue exclusion, or by analysis of cleaved PARP.

### Expression analyses

Microarray data were extracted from GEO accessions GSE45666 and GSE23978 and then normalized to the geometric median.^90, 91^

For qRT–PCR, total RNA was extracted and mRNA and miR levels were analyzed as previously described.^36^ Reactions were normalized to *B2M* or *RPLP0* for mRNA analysis, or to U6 snRNA for miR analysis. Primer sequences are listed in Supplementary Table S2. PCR reactions were performed on a Mx3005P Real-Time PCR System (Stratagene, La Jolla, CA, USA). mRNA and miR levels were determined by the ΔΔC_T_ method.^92^ For all RNA measurements, three independent experiments were performed in duplicate manner.

### Statistical analysis

Data were analyzed using either the unpaired *t*-test (two-tailed), or else one-way analysis of variance followed by Tukey's multiple comparison *ad hoc* post-test. Tumor volumes were analyzed using two-way analysis of variance with a Bonferroni post-test. Tumor initiation was analyzed using a 2 × 2 contingency table with a Fisher's exact test. Statistical analyses were performed in GraphPad Prism 5 (GraphPad Software, La Jolla, CA, USA. Differences were considered significant when the analysis yielded *P*<0.05.

## References

[bib1] 1Reya T, Morrison SJ, Clarke MF, Weissman IL. Stem cells, cancer, and cancer stem cells. Nature 2001; 414: 105–111. 11689955 10.1038/35102167

[bib2] 2Hwang-Verslues WW, Chang KJ, Lee EY, Lee WH. Breast cancer stem cells and tumor suppressor genes. J Formos Med Assoc 2008; 107: 751–766. 18926942 10.1016/S0929-6646(08)60188-6

[bib3] 3Visvader JE, Lindeman GJ. Cancer stem cells in solid tumours: accumulating evidence and unresolved questions. Nat Rev Cancer 2008; 8: 755–768. 18784658 10.1038/nrc2499

[bib4] 4Shackleton M, Quintana E, Fearon ER, Morrison SJ. Heterogeneity in cancer: cancer stem cells versus clonal evolution. Cell 2009; 138: 822–829. 19737509 10.1016/j.cell.2009.08.017

[bib5] 5Clevers H. The cancer stem cell: premises, promises and challenges. Nat Med 2011; 17: 313–319. 21386835 10.1038/nm.2304

[bib6] 6Beck B, Blanpain C. Unravelling cancer stem cell potential. Nat Rev Cancer 2013; 13: 727–738. 24060864 10.1038/nrc3597

[bib7] 7Dontu G, Abdallah WM, Foley JM, Jackson KW, Clarke MF, Kawamura MJ et al. *In vitro* propagation and transcriptional profiling of human mammary stem/progenitor cells. Genes Dev 2003; 17: 1253–1270. 12756227 10.1101/gad.1061803 PMC196056

[bib8] 8Dontu G, Al-Hajj M, Abdallah WM, Clarke MF, Wicha MS. Stem cells in normal breast development and breast cancer. Cell Prolif 2003; 36(Suppl 1): 59–72. 14521516 10.1046/j.1365-2184.36.s.1.6.x PMC6495427

[bib9] 9Al-Hajj M, Wicha MS, Benito-Hernandez A, Morrison SJ, Clarke MF. Prospective identification of tumorigenic breast cancer cells. Proc Natl Acad Sci USA 2003; 100: 3983–3988. 12629218 10.1073/pnas.0530291100 PMC153034

[bib10] 10Shipitsin M, Campbell LL, Argani P, Weremowicz S, Bloushtain-Qimron N, Yao J et al. Molecular definition of breast tumor heterogeneity. Cancer Cell 2007; 11: 259–273. 17349583 10.1016/j.ccr.2007.01.013

[bib11] 11Ginestier C, Hur MH, Charafe-Jauffret E, Monville F, Dutcher J, Brown M et al. ALDH1 is a marker of normal and malignant human mammary stem cells and a predictor of poor clinical outcome. Cell Stem Cell 2007; 1: 555–567. 18371393 10.1016/j.stem.2007.08.014 PMC2423808

[bib12] 12Hwang-Verslues WW, Kuo WH, Chang PH, Pan CC, Wang HH, Tsai ST et al. Multiple lineages of human breast cancer stem/progenitor cells identified by profiling with stem cell markers. PLoS ONE 2009; 4: e8377. 20027313 10.1371/journal.pone.0008377 PMC2793431

[bib13] 13Reya T, Clevers H. Wnt signalling in stem cells and cancer. Nature 2005; 434: 843–850. 15829953 10.1038/nature03319

[bib14] 14Mani SA, Guo W, Liao MJ, Eaton EN, Ayyanan A, Zhou AY et al. The epithelial-mesenchymal transition generates cells with properties of stem cells. Cell 2008; 133: 704–715. 18485877 10.1016/j.cell.2008.03.027 PMC2728032

[bib15] 15Karamboulas C, Ailles L. Developmental signaling pathways in cancer stem cells of solid tumors. Biochim Biophys Acta 2013; 1830: 2481–2495. 23196196 10.1016/j.bbagen.2012.11.008

[bib16] 16Rowland BD, Bernards R, Peeper DS. The KLF4 tumour suppressor is a transcriptional repressor of p53 that acts as a context-dependent oncogene. Nat Cell Biol 2005; 7: 1074–1082. 16244670 10.1038/ncb1314

[bib17] 17Rowland BD, Peeper DS. KLF4, p21 and context-dependent opposing forces in cancer. Nat Rev Cancer 2006; 6: 11–23. 16372018 10.1038/nrc1780

[bib18] 18Takahashi K, Yamanaka S. Induction of pluripotent stem cells from mouse embryonic and adult fibroblast cultures by defined factors. Cell 2006; 126: 663–676. 16904174 10.1016/j.cell.2006.07.024

[bib19] 19Takahashi K, Tanabe K, Ohnuki M, Narita M, Ichisaka T, Tomoda K et al. Induction of pluripotent stem cells from adult human fibroblasts by defined factors. Cell 2007; 131: 861–872. 18035408 10.1016/j.cell.2007.11.019

[bib20] 20McConnell BB, Yang VW. Mammalian kruppel-like factors in health and diseases. Physiol Rev 2010; 90: 1337–1381. 20959618 10.1152/physrev.00058.2009 PMC2975554

[bib21] 21McCormick SM, Eskin SG, McIntire LV, Teng CL, Lu CM, Russell CG et al. DNA microarray reveals changes in gene expression of shear stressed human umbilical vein endothelial cells. Proc Natl Acad Sci USA 2001; 98: 8955–8960. 11481467 10.1073/pnas.171259298 PMC55355

[bib22] 22Pedersen TX, Leethanakul C, Patel V, Mitola D, Lund LR, Dano K et al. Laser capture microdissection-based *in vivo* genomic profiling of wound keratinocytes identifies similarities and differences to squamous cell carcinoma. Oncogene 2003; 22: 3964–3976. 12813470 10.1038/sj.onc.1206614

[bib23] 23Yoon HS, Chen X, Yang VW. Kruppel-like factor 4 mediates p53-dependent G1/S cell cycle arrest in response to DNA damage. J Biol Chem 2003; 278: 2101–2105. 12427745 10.1074/jbc.M211027200 PMC2229830

[bib24] 24Liu Y, Sinha S, McDonald OG, Shang Y, Hoofnagle MH, Owens GK. Kruppel-like factor 4 abrogates myocardin-induced activation of smooth muscle gene expression. J Biol Chem 2005; 280: 9719–9727. 15623517 10.1074/jbc.M412862200

[bib25] 25Liu Y, Wang J, Yi Y, Zhang H, Liu J, Liu M et al. Induction of KLF4 in response to heat stress. Cell Stress Chaperones 2006; 11: 379–389. 17278886 10.1379/CSC-210.1 PMC1712684

[bib26] 26Ghaleb AM, Katz JP, Kaestner KH, Du JX, Yang VW. Kruppel-like factor 4 exhibits antiapoptotic activity following gamma-radiation-induced DNA damage. Oncogene 2007; 26: 2365–2373. 17016435 10.1038/sj.onc.1210022 PMC2230633

[bib27] 27Hamik A, Lin Z, Kumar A, Balcells M, Sinha S, Katz J et al. Kruppel-like factor 4 regulates endothelial inflammation. J Biol Chem 2007; 282: 13769–13779. 17339326 10.1074/jbc.M700078200

[bib28] 28Liao X, Haldar SM, Lu Y, Jeyaraj D, Paruchuri K, Nahori M et al. Kruppel-like factor 4 regulates pressure-induced cardiac hypertrophy. J Mol Cell Cardiol 2010; 49: 334–338. 20433848 10.1016/j.yjmcc.2010.04.008 PMC2885477

[bib29] 29Foster KW, Ren S, Louro ID, Lobo-Ruppert SM, Kie-Bell P, Grizzle W et al. Oncogene expression cloning by retroviral transduction of adenovirus E1A-immortalized rat kidney RK3E cells: transformation of a host with epithelial features by c-MYC and the zinc finger protein GKLF. Cell Growth Differ 1999; 10: 423–434. 10392904

[bib30] 30Foster KW, Liu Z, Nail CD, Li X, Fitzgerald TJ, Bailey SK et al. Induction of KLF4 in basal keratinocytes blocks the proliferation-differentiation switch and initiates squamous epithelial dysplasia. Oncogene 2005; 24: 1491–1500. 15674344 10.1038/sj.onc.1208307 PMC1361530

[bib31] 31Leng Z, Tao K, Xia Q, Tan J, Yue Z, Chen J et al. Krüppel-like factor 4 acts as an oncogene in colon cancer stem cell-enriched spheroid cells. PLoS ONE 2013; 8: e56082. 23418515 10.1371/journal.pone.0056082 PMC3572033

[bib32] 32Pandya AY, Talley LI, Frost AR, Fitzgerald TJ, Trivedi V, Chakravarthy M et al. Nuclear localization of KLF4 is associated with an aggressive phenotype in early-stage breast cancer. Clin Cancer Res 2004; 10: 2709–2719. 15102675 10.1158/1078-0432.ccr-03-0484

[bib33] 33Chu PY, Hsu NC, Liao AT, Yeh KT, Hou MF, Liu CH et al. Elevated Kruppel-like factor 4 transcription factor in canine mammary carcinoma. BMC Vet Res 2011; 7: 58. 21978458 10.1186/1746-6148-7-58 PMC3198687

[bib34] 34Kamalakaran S, Varadan V, Giercksky Russnes HE, Levy D, Kendall J, Janevski A et al. DNA methylation patterns in luminal breast cancers differ from non-luminal subtypes and can identify relapse risk independent of other clinical variables. Mol Oncol 2011; 5: 77–92. 21169070 10.1016/j.molonc.2010.11.002 PMC5528275

[bib35] 35Chen CJ, Lin SE, Lin YM, Lin SH, Chen DR, Chen CL et al. Association of expression of kruppel-like factor 4 and kruppel-like factor 5 with the clinical manifestations of breast cancer. Pathol Oncol Res 2012; 18: 161–168. 21674249 10.1007/s12253-011-9422-7

[bib36] 36Lin CC, Liu LZ, Addison JB, Ivanov AV, Ruppert JM. A KLF4-miRNA-206 autoregulatory feedback loop can promote or inhibit protein translation depending upon cell context. Mol Cell Biol 2011; 31: 2513–2527. 21518959 10.1128/MCB.01189-10 PMC3133414

[bib37] 37Sharma SB, Lin CC, Farrugia MK, McLaughlin SL, Ellis EJ, Brundage KM et al. microRNAs-206 and -21 cooperate to promote RAS-ERK signaling by suppressing the translation of RASA1 and SPRED1. Mol Cell Biol 2014; 34: 4143–4164. 25202123 10.1128/MCB.00480-14 PMC4248710

[bib38] 38Anderson C, Catoe H, Werner R. MIR-206 regulates connexin43 expression during skeletal muscle development. Nucleic Acids Res 2006; 34: 5863–5871. 17062625 10.1093/nar/gkl743 PMC1635318

[bib39] 39Reynolds BA, Weiss S. Clonal and population analyses demonstrate that an EGF-responsive mammalian embryonic CNS precursor is a stem cell. Dev Biol 1996; 175: 1–13. 8608856 10.1006/dbio.1996.0090

[bib40] 40Uchida N, Buck DW, He D, Reitsma MJ, Masek M, Phan TV et al. Direct isolation of human central nervous system stem cells. Proc Natl Acad Sci USA 2000; 97: 14720–14725. 11121071 10.1073/pnas.97.26.14720 PMC18985

[bib41] 41Dontu G, Wicha MS. Survival of mammary stem cells in suspension culture: implications for stem cell biology and neoplasia. J Mammary Gland Biol Neoplasia 2005; 10: 75–86. 15886888 10.1007/s10911-005-2542-5

[bib42] 42Harrison H, Farnie G, Howell SJ, Rock RE, Stylianou S, Brennan KR et al. Regulation of breast cancer stem cell activity by signaling through the Notch4 receptor. Cancer Res 2010; 70: 709–718. 20068161 10.1158/0008-5472.CAN-09-1681 PMC3442245

[bib43] 43Chaffer CL, Brueckmann I, Scheel C, Kaestli AJ, Wiggins PA, Rodrigues LO et al. Normal and neoplastic nonstem cells can spontaneously convert to a stem-like state. Proc Natl Acad Sci USA 2011; 108: 7950–7955. 21498687 10.1073/pnas.1102454108 PMC3093533

[bib44] 44Iorio MV, Ferracin M, Liu CG, Veronese A, Spizzo R, Sabbioni S et al. MicroRNA gene expression deregulation in human breast cancer. Cancer Res 2005; 65: 7065–7070. 16103053 10.1158/0008-5472.CAN-05-1783

[bib45] 45Kondo N, Toyama T, Sugiura H, Fujii Y, Yamashita H. miR-206 Expression is down-regulated in estrogen receptor alpha-positive human breast cancer. Cancer Res 2008; 68: 5004–5008. 18593897 10.1158/0008-5472.CAN-08-0180

[bib46] 46Herschkowitz JI, Simin K, Weigman VJ, Mikaelian I, Usary J, Hu Z et al. Identification of conserved gene expression features between murine mammary carcinoma models and human breast tumors. Genome Biol 2007; 8: R76. 17493263 10.1186/gb-2007-8-5-r76 PMC1929138

[bib47] 47Honeth G, Bendahl PO, Ringner M, Saal LH, Gruvberger-Saal SK, Lovgren K et al. The CD44+/CD24- phenotype is enriched in basal-like breast tumors. Breast Cancer Res 2008; 10: R53. 18559090 10.1186/bcr2108 PMC2481503

[bib48] 48Creighton CJ, Li X, Landis M, Dixon JM, Neumeister VM, Sjolund A et al. Residual breast cancers after conventional therapy display mesenchymal as well as tumor-initiating features. Proc Natl Acad Sci USA 2009; 106: 13820–13825. 19666588 10.1073/pnas.0905718106 PMC2720409

[bib49] 49Perou CM. Molecular stratification of triple-negative breast cancers. Oncologist 2010; 15(Suppl 5): 39–48. 21138954 10.1634/theoncologist.2010-S5-39

[bib50] 50Ibarra I, Erlich Y, Muthuswamy SK, Sachidanandam R, Hannon GJ. A role for microRNAs in maintenance of mouse mammary epithelial progenitor cells. Genes Dev 2007; 21: 3238–3243. 18079172 10.1101/gad.1616307 PMC2113025

[bib51] 51Charafe-Jauffret E, Ginestier C, Iovino F, Wicinski J, Cervera N, Finetti P et al. Breast cancer cell lines contain functional cancer stem cells with metastatic capacity and a distinct molecular signature. Cancer Res 2009; 69: 1302–1313. 19190339 10.1158/0008-5472.CAN-08-2741 PMC2819227

[bib52] 52Luo M, Fan H, Nagy T, Wei H, Wang C, Liu S et al. Mammary epithelial-specific ablation of the focal adhesion kinase suppresses mammary tumorigenesis by affecting mammary cancer stem/progenitor cells. Cancer Res 2009; 69: 466–474. 19147559 10.1158/0008-5472.CAN-08-3078 PMC3039129

[bib53] 53Fillmore CM, Kuperwasser C. Human breast cancer cell lines contain stem-like cells that self-renew, give rise to phenotypically diverse progeny and survive chemotherapy. Breast Cancer Res 2008; 10: R25. 18366788 10.1186/bcr1982 PMC2397524

[bib54] 54Park SY, Lee HE, Li H, Shipitsin M, Gelman R, Polyak K. Heterogeneity for stem cell-related markers according to tumor subtype and histologic stage in breast cancer. Clin Cancer Res 2010; 16: 876–887. 20103682 10.1158/1078-0432.CCR-09-1532 PMC2818503

[bib55] 55Giatromanolaki A, Sivridis E, Fiska A, Koukourakis MI. The CD44+/CD24- phenotype relates to 'triple-negative' state and unfavorable prognosis in breast cancer patients. Med Oncol 2011; 28: 745–752. 20405247 10.1007/s12032-010-9530-3

[bib56] 56Lu TP, Lee CY, Tsai MH, Chiu YC, Hsiao CK, Lai LC et al. miRSystem: an integrated system for characterizing enriched functions and pathways of microRNA targets. PLoS ONE 2012; 7: e42390. 22870325 10.1371/journal.pone.0042390 PMC3411648

[bib57] 57Afonja O, Juste D, Das S, Matsuhashi S, Samuels HH. Induction of PDCD4 tumor suppressor gene expression by RAR agonists, antiestrogen and HER-2/neu antagonist in breast cancer cells. Evidence for a role in apoptosis. Oncogene 2004; 23: 8135–8145. 15361828 10.1038/sj.onc.1207983

[bib58] 58Lankat-Buttgereit B, Goke R. The tumour suppressor Pdcd4: recent advances in the elucidation of function and regulation. Biol Cell 2009; 101: 309–317. 19356152 10.1042/BC20080191

[bib59] 59Santhanam AN, Baker AR, Hegamyer G, Kirschmann DA, Colburn NH. Pdcd4 repression of lysyl oxidase inhibits hypoxia-induced breast cancer cell invasion. Oncogene 2010; 29: 3921–3932. 20498644 10.1038/onc.2010.158 PMC3419530

[bib60] 60Vlachos IS, Kostoulas N, Vergoulis T, Georgakilas G, Reczko M, Maragkakis M et al. DIANA miRPath v.2.0: investigating the combinatorial effect of microRNAs in pathways. Nucleic Acids Res 2012; 40: W498–W504. 22649059 10.1093/nar/gks494 PMC3394305

[bib61] 61Kim HK, Lee YS, Sivaprasad U, Malhotra A, Dutta A. Muscle-specific microRNA miR-206 promotes muscle differentiation. J Cell Biol 2006; 174: 677–687. 16923828 10.1083/jcb.200603008 PMC2064311

[bib62] 62Laird DW, Fistouris P, Batist G, Alpert L, Huynh HT, Carystinos GD et al. Deficiency of connexin43 gap junctions is an independent marker for breast tumors. Cancer Res 1999; 59: 4104–4110. 10463615

[bib63] 63Qin H, Shao Q, Curtis H, Galipeau J, Belliveau DJ, Wang T et al. Retroviral delivery of connexin genes to human breast tumor cells inhibits *in vivo* tumor growth by a mechanism that is independent of significant gap junctional intercellular communication. J Biol Chem 2002; 277: 29132–29138. 12042301 10.1074/jbc.M200797200

[bib64] 64Ponti D, Costa A, Zaffaroni N, Pratesi G, Petrangolini G, Coradini D et al. Isolation and *in vitro* propagation of tumorigenic breast cancer cells with stem/progenitor cell properties. Cancer Res 2005; 65: 5506–5511. 15994920 10.1158/0008-5472.CAN-05-0626

[bib65] 65Shao Q, Wang H, McLachlan E, Veitch GI, Laird DW. Down-regulation of Cx43 by retroviral delivery of small interfering RNA promotes an aggressive breast cancer cell phenotype. Cancer Res 2005; 65: 2705–2711. 15805269 10.1158/0008-5472.CAN-04-2367

[bib66] 66McLachlan E, Shao Q, Laird DW. Connexins and gap junctions in mammary gland development and breast cancer progression. J Membr Biol 2007; 218: 107–121. 17661126 10.1007/s00232-007-9052-x

[bib67] 67Li Z, Zhou Z, Welch DR, Donahue HJ. Expressing connexin 43 in breast cancer cells reduces their metastasis to lungs. Clin Exp Metastasis 2008; 25: 893–901. 18839320 10.1007/s10585-008-9208-9 PMC2754227

[bib68] 68Plante I, Stewart MK, Barr K, Allan AL, Laird DW. Cx43 suppresses mammary tumor metastasis to the lung in a Cx43 mutant mouse model of human disease. Oncogene 2011; 30: 1681–1692. 21151177 10.1038/onc.2010.551

[bib69] 69Dick JE. Stem cell concepts renew cancer research. Blood 2008; 112: 4793–4807. 19064739 10.1182/blood-2008-08-077941

[bib70] 70Sirnes S, Bruun J, Kolberg M, Kjenseth A, Lind GE, Svindland A et al. Connexin43 acts as a colorectal cancer tumor suppressor and predicts disease outcome. Int J Cancer 2012; 131: 570–581. 21866551 10.1002/ijc.26392

[bib71] 71Yu F, Yao H, Zhu P, Zhang X, Pan Q, Gong C et al. let-7 regulates self renewal and tumorigenicity of breast cancer cells. Cell 2007; 131: 1109–1123. 18083101 10.1016/j.cell.2007.10.054

[bib72] 72Shimono Y, Zabala M, Cho RW, Lobo N, Dalerba P, Qian D et al. Downregulation of miRNA-200c links breast cancer stem cells with normal stem cells. Cell 2009; 138: 592–603. 19665978 10.1016/j.cell.2009.07.011 PMC2731699

[bib73] 73Hwang-Verslues WW, Chang PH, Wei PC, Yang CY, Huang CK, Kuo WH et al. miR-495 is upregulated by E12/E47 in breast cancer stem cells, and promotes oncogenesis and hypoxia resistance via downregulation of E-cadherin and REDD1. Oncogene 2011; 30: 2463–2474. 21258409 10.1038/onc.2010.618

[bib74] 74Deng L, Shang L, Bai S, Chen J, He X, Martin-Trevino R et al. MicroRNA100 inhibits self-renewal of breast cancer stem-like cells and breast tumor development. Cancer Res 2014; 74: 6648–6660. 25217527 10.1158/0008-5472.CAN-13-3710 PMC4370193

[bib75] 75McCarthy JJ. MicroRNA-206: the skeletal muscle-specific myomiR. Biochim Biophys Acta 2008; 1779: 682–691. 18381085 10.1016/j.bbagrm.2008.03.001 PMC2656394

[bib76] 76Williams AH, Valdez G, Moresi V, Qi X, McAnally J, Elliott JL et al. MicroRNA-206 delays ALS progression and promotes regeneration of neuromuscular synapses in mice. Science 2009; 326: 1549–1554. 20007902 10.1126/science.1181046 PMC2796560

[bib77] 77Cacchiarelli D, Martone J, Girardi E, Cesana M, Incitti T, Morlando M et al. MicroRNAs involved in molecular circuitries relevant for the Duchenne muscular dystrophy pathogenesis are controlled by the dystrophin/nNOS pathway. Cell Metab 2010; 12: 341–351. 20727829 10.1016/j.cmet.2010.07.008

[bib78] 78Dey BK, Gagan J, Dutta A. miR-206 and -486 induce myoblast differentiation by downregulating Pax7. Mol Cell Biol 2011; 31: 203–214. 21041476 10.1128/MCB.01009-10 PMC3019853

[bib79] 79Liu N, Williams AH, Maxeiner JM, Bezprozvannaya S, Shelton JM, Richardson JA et al. microRNA-206 promotes skeletal muscle regeneration and delays progression of Duchenne muscular dystrophy in mice. J Clin Invest 2012; 122: 2054–2065. 22546853 10.1172/JCI62656 PMC3366415

[bib80] 80Tavazoie SF, Alarcon C, Oskarsson T, Padua D, Wang Q, Bos PD et al. Endogenous human microRNAs that suppress breast cancer metastasis. Nature 2008; 451: 147–152. 18185580 10.1038/nature06487 PMC2782491

[bib81] 81Zhang T, Liu M, Wang C, Lin C, Sun Y, Jin D et al. Down-regulation of MiR-206 promotes proliferation and invasion of laryngeal cancer by regulating VEGF expression. Anticancer Res 2011; 31: 3859–3863. 22110210

[bib82] 82Chen X, Yan Q, Li S, Zhou L, Yang H, Yang Y et al. Expression of the tumor suppressor miR-206 is associated with cellular proliferative inhibition and impairs invasion in ERalpha-positive endometrioid adenocarcinoma. Cancer Lett 2012; 314: 41–53. 21983130 10.1016/j.canlet.2011.09.014

[bib83] 83Zhou J, Tian Y, Li J, Lu B, Sun M, Zou Y et al. miR-206 is down-regulated in breast cancer and inhibits cell proliferation through the up-regulation of cyclinD2. Biochem Biophys Res Commun 2013; 433: 207–212. 23466356 10.1016/j.bbrc.2013.02.084

[bib84] 84Ma L, Reinhardt F, Pan E, Soutschek J, Bhat B, Marcusson EG et al. Therapeutic silencing of miR-10b inhibits metastasis in a mouse mammary tumor model. Nat Biotechnol 2010; 28: 341–347. 20351690 10.1038/nbt.1618 PMC2852471

[bib85] 85Kasinski AL, Slack FJ. Epigenetics and genetics. MicroRNAs en route to the clinic: progress in validating and targeting microRNAs for cancer therapy. Nat Rev Cancer 2011; 11: 849–864. 22113163 10.1038/nrc3166 PMC4314215

[bib86] 86Kasinski AL, Slack FJ. Arresting the culprit: targeted antagomir delivery to sequester oncogenic miR-221 in HCC. Mol Ther Nucleic Acids 2012; 1: e12. 23343881 10.1038/mtna.2012.2 PMC3381591

[bib87] 87Babar IA, Cheng CJ, Booth CJ, Liang X, Weidhaas JB, Saltzman WM et al. Nanoparticle-based therapy in an *in vivo* microRNA-155 (miR-155)-dependent mouse model of lymphoma. Proc Natl Acad Sci USA 2012; 109: E1695–E1704. 22685206 10.1073/pnas.1201516109 PMC3387084

[bib88] 88Janssen HL, Reesink HW, Lawitz EJ, Zeuzem S, Rodriguez-Torres M, Patel K et al. Treatment of HCV infection by targeting microRNA. N Engl J Med 2013; 368: 1685–1694. 23534542 10.1056/NEJMoa1209026

[bib89] 89Zuker M. Mfold web server for nucleic acid folding and hybridization prediction. Nucleic Acids Res 2003; 31: 3406–3415. 12824337 10.1093/nar/gkg595 PMC169194

[bib90] 90Zhu M, Yi M, Kim CH, Deng C, Li Y, Medina D et al. Integrated miRNA and mRNA expression profiling of mouse mammary tumor models identifies miRNA signatures associated with mammary tumor lineage. Genome Biol 2011; 12: R77. 21846369 10.1186/gb-2011-12-8-r77 PMC3245617

[bib91] 91Lee CH, Kuo WH, Lin CC, Oyang YJ, Huang HC, Juan HF et al. MicroRNA-regulated protein-protein interaction networks and their functions in breast cancer. Int J Mol Sci 2013; 14: 11560–11606. 23722663 10.3390/ijms140611560 PMC3709748

[bib92] 92Chen C, Ridzon DA, Broomer AJ, Zhou Z, Lee DH, Nguyen JT et al. Real-time quantification of microRNAs by stem-loop RT-PCR. Nucleic Acids Res 2005; 33: e179. 16314309 10.1093/nar/gni178 PMC1292995

