# Supplementary material for: Kruppel-like factor 4 signals through microRNA-206 to promote tumor initiation and cell survival
Source: Oncogenesis. 2015 Jun 8;4(6):e155–. doi: 10.1038/oncsis.2015.8 (PMC4753526; doi:10.1038/oncsis.2015.8)
Supplement: Supplementary Tables [file oncsis20158x1.pdf]

Table S1: Oligonucleotides for PCR mutagenesis

Gene	Sense Primer (5'-3')*	Antisense Primer (5'-3')*
PDCD4-mt206-A	GCCATGTTTATTATCTAATacTctCAAGTT TTGCATTGATGTCTG	CAGACATCAATGCAAAACTTGagAgtATT AGATAATAAACATGGC
PDCD4-mt206-B	AATATGTAATACCTTCCATacTctCATCAT CCTTAAATTCTGTTA	TAACAGAATTTAAGGATGATGagAgtATG GAAGGTATTACATATT
CX43-mt206-A	CCTTAAGTCCCTGCTAAAAacTctCATTGT TAAAATTTGCACTTT	AAAGTGCAAATTTTAACAATGagAgtTTT TAGCAGGGACTTAAGG

*Lower case indicates mutated nucleotides.

Table S2: Primers for real-time quantitative PCR analysis

Gene	Sense Primer (5'-3')	Antisense Primer (5'-3')
KLF4	AGAGTTCCCATCTCAAGGCA	GTCAGTTCATCTGAGCGGG
CD44	TGCCGCTTTGCAGGTGTAT	GGCCTCCGTCCGAGAGA
CD24	AAACAACAACTGGAACTTCAAGTAACT	GGTGGTGGCATTAGTTGGATTT
c-MYC	CGACGAGACCTTCATCAAAA	TGCTGTCGTTGAGAGGGTAG
SOX2	AACCCCAAGATGCACAACTC	GCTTAGCCTCGTCGATGAAC
OCT3/4	GAAGCAGAAGAGGATCACCTTG	TTCTTAAGGCTGAGCTGCAAG
NANOG	AATACCTCAGCCTCCAGCAGATG	TGCGTCACACCATTGCTATTCTTC
ZEB1	GCACAACCAAGTGCAGAAGA	CATTTGCAGATTGAGGCTGA
ZEB2	GCCATCTGATCCGCTCTTATC	ACCTGTGTCCACTACATTGTC
SNAI1	TCTGAGTGGGTCTGGAGGTG	CTCTAGGCCCTGGCTGCTAC
SNAI2	AGCATTTCAACGCCTCCA	GGATCTCTGGTTGTGGTATGAC
CX43	AGGAGTTCAATCACTTGGCG	GAGTTTGCCTAAGGCGCTC
PDCD4	TGCAAGCGAAATTAAGGGAA	TCCTCAGTCCCAGCATTTTC
B2M	TGCTCGCGCTACTCTCTCTTT	TCTGCTGGATGACGTGAGTAAAC
RPLP0	GGACCCGAGAAGACCTCCTT	GCACATCACTCAGAATTTCAATGG
